# EASIX (endothelial activation and stress index) predicts mortality in patients with coronary artery disease

**DOI:** 10.1007/s00392-024-02534-y

**Published:** 2024-09-10

**Authors:** Daniel Finke, Hauke Hund, Norbert Frey, Thomas Luft, Lorenz H. Lehmann

**Affiliations:** 1 Department of Cardiology, Medizinische Klinik III, University Hospital Heidelberg, Im Neuenheimer Feld 410, 69120 Heidelberg, Germany; 2 German Center for Cardiovascular Research (DZHK), Partnersite Heidelberg/Mannheim, Heidelberg, Germany; 3 Department of Oncology and Haematology, Medizinische Klinik V, University Hospital Heidelberg, Im Neuenheimer Feld 410, 69120 Heidelberg, Germany; 4 German Cancer Research Center (DKFZ), 69120 Heidelberg, Germany

**Keywords:** Endothelial activation and stress index, EASIX, Coronary artery disease, Heart failure, Left ventricular ejection fraction, LVEF, Mortality prediction, Endothelial dysfunction, Allogeneic stem cell transplantation, Transplant-associated thrombotic microangiopathy, TA-TMA, Creatinine, Lactate dehydrogenase, LDH, Platelets, Thrombocytes, Hs-cTnT, NT-proBNP

## Abstract

**Background:**

Coronary interventions reduce morbidity and mortality in patients with acute coronary syndrome. However, the risk of mortality for patients with coronary artery disease (CAD) additionally depends on their systemic endothelial health status. The ‘Endothelial Activation and Stress Index’ (EASIX) predicts endothelial complications and survival in diverse clinical settings.

**Objective:**

We hypothesized that EASIX may predict mortality in patients with CAD.

**Methods:**

In 1283 patients undergoing coronary catheterization (CC) and having a diagnosis of CAD, EASIX was measured within 52 days (range − 1 year to − 14 days) before CC and correlated with overall survival. In an independent validation cohort of 1934 patients, EASIXval was measured within 174 days (+ 28 days to + 11 years) after CC.

**Results:**

EASIX predicted the risk of mortality after CC (per log2: hazard ratio (HR) 1.29, 95% confidence interval: [1.18–1.41], *p* < 0.001) in multivariable Cox regression analyses adjusting for age, sex, a high-grade coronary stenosis ≥ 90%, left ventricular ejection fraction, arterial hypertension and diabetes. In the independent cohort, EASIX correlated with EASIXval with rho = 0.7. The long-term predictive value of EASIXval was confirmed (per log2: HR 1.53, [1.42–1.64], *p* < 0.001) and could be validated by integrated Brier score and concordance index. Pre-established cut-offs (0.88–2.32) associated with increased mortality (cut-off 0.88: HR training: 1.63; HR validation: 1.67, *p* < 0.0001 and cut-off 2.32: HR training: 3.57; HR validation: 4.65, *p* < 0.0001).

**Conclusions:**

We validated EASIX as a potential biomarker to predict death of CAD patients, irrespective of the timing either before or after catheterization.

**Graphical abstract:**

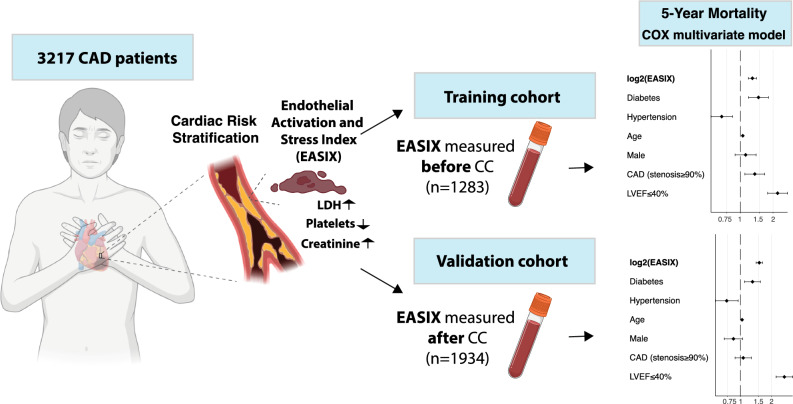

**Supplementary Information:**

The online version contains supplementary material available at 10.1007/s00392-024-02534-y.

## Introduction

The term coronary artery disease (CAD) usually refers to the localized macrovascular stenosis; however, the disease is associated with macro- and microvascular defects throughout the entire organism [1–3]. In patients with unstable angina and acute coronary syndrome (ACS), early cardiac interventions reduce the mortality in the acute setting. Stable CAD patients however, who exhibit elevated mortality rates, are notably more challenging to treat [4]. Results of the ORBITA and ISCHEMIA trials showed that percutaneous coronary intervention (PCI) does not substantially alleviate symptoms of stable angina pectoris, nor does it enhance exercise capabilities, nor does it prevent acute myocardial infarction (AMI) or death in such patients [5, 6]. In addition to modifiable CAD risk factors, cardiac microvascular dysfunction (CMD) [7, 8], retinal vascular signs [9] and renal endothelial dysfunction [10], associate with poor outcome in CAD patients. CMD is increasingly recognized as a systemic disorder with commonly used biomarkers in heart, brain and kidney associated complications [11]. The role of CMD is explicitly examined in patient cohorts with heart failure with preserved ejection fraction (HFpEF) and is associated with the elevations of biomarkers, such as B-Type Natriuretic Peptide (NT-proBNP), or other cardiac co-morbidities, such as obesity, diabetes and atrial fibrillation [12, 13].

Cardiac biomarkers such as cardiac troponin or NT-proBNP are established well in the prediction of overall mortality of CAD patients. Even though endothelial dysfunction is not directly linked to damaged cardiomyocytes, cardiac troponins are elevated in patients with coronary endothelial dysfunction. This might indicate subclinical cardiac ischemia, associated with increased mortality [14]. Until now, there is no validated, easily accessible biomarker of endothelial dysfunction with prognostic value in CAD patients.

Endothelial dysfunction is a common link between cardiology and a variety of other specialties, e.g., hematology. Endothelial complications are strongly associated with mortality of hematological patients, in particular after allogeneic stem cell transplantation (alloSCT) [15]. Endothelial-related complications after alloSCT include transplant-associated thrombotic microangiopathy (TAM), sinusoidal obstruction syndrome/veno-occlusive disease, refractory acute graft-versus-host disease (GVHD), acute and chronic kidney failure, microangiopathy in sepsis-associated cardiovascular failure, and late complications of macrovascular disease [16, 17]. Outside alloSCT, mortality of several malignant diseases was shown to be associated with endothelial markers, including multiple myeloma [18], small cell lung cancer [19] or urothelial cell carcinoma [20] and lower-risk myelodysplastic syndromes (MDS) [21]. Lower risk MDS patients have high rates of cardiovascular mortality and share characteristics with clonal hematopoiesis of indeterminate potential (CHIP), which itself was recognized as a risk factor for atherosclerosis [22], progression of ischemic heart disease [23] and for major adverse cardiac events in patients with CMD [24].

The urgent need to predict endothelial complications after alloSCT early led us to develop EASIX (Endothelial Activation and Stress Index) that includes the three relevant routine lab markers required to diagnose TAM: [creatinine x LDH]/platelets). As in all manifestations of atypical hemolytic uremic syndromes (aHUS) [25], these three peripheral blood markers show typical changes also in patients with TAM: a rise in LDH and creatinine and a decline in platelet counts [26]. The EASIX formula integrates these changes into a value that represents the three markers in one continuous function. EASIX allows predicting the risk of endothelial complications such as TAM after alloSCT as early as before starting chemotherapy for alloSCT [27].

Over the last years, EASIX was established and validated as a biomarker to predict endothelial dysfunction and survival in patients after alloSCT and after GVHD [27, 28] and to predict sepsis already prior to conditioning therapy [29, 30]. Further, EASIX can be used to predict mortality due to sepsis after admission to the ICU [31], mortality of severe liver diseases [32], and mortality and severe courses of COVID-19 at admission to hospital [33].

The strong association of EASIX with mortality in any context investigated so far led us to hypothesize that EASIX might be a global marker of endothelial cell dysfunction and is also able to predict outcome of CAD patients.

Therefore, this study investigates EASIX in two independent cohorts of patients with CAD. In the first cohort, EASIX scores were measured at median 52 days (range 14–365) before coronary angiography (EASIX). This assumes independency of acute events that are directly leading to coronary angiography (e.g., myocardial infarction). In this cohort, EASIX was then correlated with overall mortality. In a validation cohort of 1934 patients, EASIX scores were measured at median 174 days (range 28–4246) after coronary angiography (EASIXval). The impact of EASIX on the 5-year outcome in the training cohort was validated in the independent validation cohort.

## Methods

### Study population and data collection

The training cohort included 1283 patients and the validation cohort included 1934 patients who underwent CC. All patients that were included in the study showed a stenosis of at least 25% in any coronary segment in CC considering the presence of CAD and were treated at University Hospital Heidelberg from January 2005 to December 2017. The patient cohorts were selected retrospectively by number and timing of the blood samples of EASIX parameters (platelet count, creatinine and LDH), available either only 52 days at median (range 14–365 days) prior cardiac catheterization (CC) or patients with only one sampling of EASIX (EASIX, training cohort). The second validation cohort was selected from patients with samples prior and after CC (median: 174 days, range 28–4246 days) (EASIXval, validation cohort). In the patients of the non-overlapping, independent validation cohort, EASIX was additionally measured before CC (median: 58 days, range: 14–365, *n* = 1934) and used to calculate the pre/post correlation. If multiple measurements were available, the nearest time point to coronary angiography was chosen.

Patient data, including epidemiological data, dates of cardiac catheterizations, laboratory values and risk factors, were gathered by an automated request of the electronic medical reports, using the cardiac Research Data Warehouse. Reports of cardiac catheterization were requested from the internal electronic catheterization database. The degree of coronary lesions per segment, if a PCI was performed and LVEF (measured via angiography) were reported. Survival data were assessed by an automated request from the respective registration offices. The date of survival assessment was January 19, 2023. The study protocol was approved by the ethic committee of the Medical Faculty of the University Heidelberg (S-286/2017). All patients were treated according to the Declaration of Helsinki.

### Laboratory and cardiac parameters

Endothelial Activation and Stress Index (EASIX) is calculated by the expression: LDH (U/L) × Creatinine (mg/dL)/Thrombocytes (per nL) [28]. LDH, creatinine and platelet counts were routinely measured at the central laboratory of University Hospital Heidelberg.

Cardiac high-sensitivity troponin T (hs-cTnT) was measured on the Elecsys system (Roche Diagnostics) at CC (median: + 0.6 days, range − 7 to + 7 days) at the central laboratory of University Hospital Heidelberg. Limit of blank (LoB), Limit of detection (LoD), 10% coefficient of variation (CV) and 99th percentile cut-off values for the hs-cTnT assay were 3, 5, 13 and 14 ng/L. NT-proBNP was measured as well at the same time as the CC (median: − 0.16 days, range − 7 to + 7 days) using the Stratus^®^ CS Acute CareTM NT‐proBNP assay (Siemens AG, Berlin and Munich, Germany). Log2-transformed hs-cTnT and NT-proBNP were used for statistical analysis. Impairments of the LVEF were classified as suggested by the current guidelines of the European Society of Cardiology (ESC). A reduced LVEF (rEF) was defined as LVEF ≤ 40% [34].

### Definitions and statistical analysis

Statistical analysis was performed in R (version 4.3.1). To compare patient data with dichotomic variables, the chi-squared test was used and an unpaired t test was applied for continuous variables. *P* values < 0.05 were considered as significant. Linear correlation was calculated via the spearman method, Rho and *p*-value were reported.

As previously reported, the log2-transformed EASIX was calculated for Cox regression analysis with continuous values [21]. In Kaplan–Meier analysis, EASIX was used in quartiles and two externally established cut-offs were applied in this study: EASIX = 2.32 is a validated threshold measured pre-alloSCT to predict risk of sepsis [29, 30] and EASIX = 0.88 is the third IQR of *n* = 47 normal individuals included in the “Endothelial Cell Dysfunction and Outcome (EndoCDO-H)” study [35].

Survival analyses were calculated by the Kaplan–Meier method within the *survival* package (version 3.5.5). Overall survival (OS) was defined as the time difference between the date of CC and the date of death. Analyzing 5-year follow-ups, patients alive were censored after the respective time difference. Cause-specific Cox regression and multinominal logistic regression models were used for adjusted survival analysis (*nnet* package, version 7.3.19). Adjustments were made to diabetes, arterial hypertension, the presence of a high-grade coronary artery stenosis (any stenosis ≥ 90% in any segment), age, sex and LVEF.

Multivariate Cox regression models were used to predict overall survival within the *survivalAnalysis* package (version 0.3.0). The *pec* package *(version 2023.4.12)* was used for Cox model performance tests, prediction errors were reported as Brier scores and concordance indexes (Harrell C-Index and Gönen & Heller K). External validation of the Cox Model of the training cohort was assessed as published by Royston et al. [36]. Model misspecification was calculated by building a new model in the validation cohort with the offset of the prognostic index that was calculated in the training cohort. A regression coefficient close to 0 describes a similar predictive value in both cohorts. Prediction of the validation cohort survival using the previously calculated multivariate Cox model of the training cohort was done via the *pec* package *(version 2023.4.12).*

## Results

### Patient characteristics

Patient characteristics of the training cohort (EASIX) and the validation cohort (EASIXval) are summarized in Table 1. In the training cohort, the median age was 71 years and 67.0% of patients were male and 28.1% of the patients were diagnosed with diabetes, 25.7% were obese and 76.3% had arterial hypertension.

**Table 1:** Patient characteristics cohort

	Overall	Training cohort (EASIX)	Validation cohort (EASIXval)	*p*-value
*N*	3217	1283	1934	
Sex (male, %)	2274 (70.7)	859 (67.0)	1415 (73.2)	0.001
Age (median [IQR])	71 [62, 78]	71 [62, 78]	70 [61, 77]	0.053
Diabetes (%)	1083 (33.7)	361 (28.1)	722 (37.3)	< 0.001
Hypertension (%)	2678 (83.2)	979 (76.3)	1699 (87.8)	< 0.001
BMI < 25 kg/m^2^ (%)	567 (36.7)	159 (33.3)	408 (38.2)	< 0.001
BMI 25–30 kg/m^2^ (%)	608 (39.3)	196 (41.0)	412 (38.6)	< 0.001
BMI > 30 kg/m^2^ (%)	371 (24.0)	123 (25.7)	248 (23.2)	0.005
CAD (stenosis ≥ 50%)	2616 (81.3)	1046 (81.5)	1570 (81.2)	0.840
CAD (stenosis ≥ 90%)	1150 (35.7)	442 (34.5)	708 (36.6)	0.225
PCI (%)	1012 (31.5)	414 (32.3)	598 (30.9)	0.443
LVEF ≤ 40% (%)	1247 (40.0)	479 (38.8)	768 (40.8)	0.176
LVEF 41–49% (%)	749 (24.0)	311 (25.2)	438 (23.3)	0.295
LVEF ≥ 50% (%)	1120 (35.9)	444 (36.0)	676 (35.9)	0.840
Pre-creatinine (median [IQR])	0.99 [0.81, 1.27]	0.96 [0.79, 1.21]	1.0 [0.84, 1.30]	< 0.001
Pre-LDH (median [IQR])	240 [200, 290]	250 [205.5, 307]	230 [197, 281]	< 0.001
Pre-Platelets (median [IQR])	230 [186, 280]	230 [188, 288]	220 [184, 273.75]	0.002
Post-Creatinine (median [IQR])	1.1 [0.86, 1.40]	–	1.1 [0.86, 1.40]	
Post-LDH (median [IQR])	240 [203, 291]	–	240 [203, 291]	
Post-Platelets (median [IQR])	220 [181, 270]	–	220 [181, 270]	
EASIX (median [IQR])	1.1 [0.74, 1.70]	1.1 [0.74, 1.73]	1.1 [0.75, 1.68]	0.843
EASIXval (median [IQR])	1.2 [0.80, 1.89]	–	1.2 [0.80, 1.89]	
hs-cTnT (median [IQR])	38 [16.90, 129.68]	48 [18, 181]	33 [16, 99]	< 0.001
NT-proBNP (median [IQR])	580 [169, 2454]	580 [139.5, 3435.5]	580 [185, 2229]	0.823
Median survival (mean (SD))	1700 (1,367.66)	1200 (1,264.57)	1900 (1,360.94)	< 0.001
5-year overall mortality (%)	923 (28.7)	396 (30.9)	527 (27.2)	0.029

*BMI* body mass index, *CAD* coronary artery disease, *EASIX* endothelial activation and stress index, *hs-cTnT* cardiac high-sensitivity troponin T*, LDH* lactate dehydrogenase, *LVEF* left ventricular function, *NT-proBNP* B-type natriuretic peptide, *PCI* percutaneous coronary intervention, *post*-values assessed 28–4246 days after cardiac catheterization, *pre*-values assessed 14–365 days before cardiac catheterizationa

In the validation cohort, the median age was 70 years, 73.2% of patients were male, 37.3% had diabetes, 23.2% were obese and 87.8% had arterial hypertension. EASIX values did not differ significantly between the training and the validation cohort (trainings cohort: median: 1.1, IQR [0.74, 1.73], validation cohort: median: 1.1, IQR [0.75, 1.68], *p* = 0.843). The overall mortality after 5 years was comparable, but slightly lower in the validation cohort (30.9% vs 27.2%, *p* = 0.029).

### EASIX measured before cardiac catheterization (EASIX, training cohort) predicts overall survival after cardiac catheterization

**Fig. 1:**
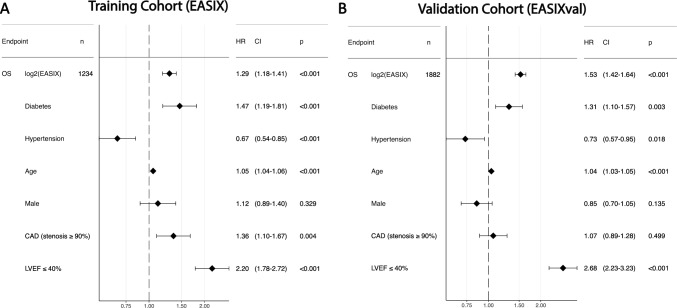
Forest plots representing Cox multivariate logistic regression model for 5-year overall mortality including log2 (EASIX) and diabetes, hypertension, age, the male sex, a high-grade coronary artery stenosis (≥ 90%) and reduced LVEF ≤ 40% as confounders in the training cohort **(A)** and the validation cohort **(B)**. *CI* confidence interval, *HR* Hazard ratio, *OS* overall survival

In the EASIX cohort creatinine, LDH and platelets were retrospectively retrieved from electronic files at a median of 52 days (range 14–364) before coronary angiography (stable clinical phase). In multivariate Cox regression analysis, including age, sex, diabetes, arterial hypertension, a high-grade coronary artery stenosis (in any segment ≥ 90%) and a reduced LVEF (LVEF ≤ 40%), log2EASIX independently predicted OS (HR: 1.29, CI 1.18–1.41, *p* < 0.001), next to diabetes (HR: 1.47, CI 1.19–1.81, *p* < 0.001), age (HR: 1.05, CI 1.04–1.06, *p* < 0.001), a high-grade coronary stenosis (HR: 1.36, CI 1.10–1.67, *p* = 0.004) and a reduced LVEF (HR: 2.20, CI 1.78–2.72, *p* < 0.001) (Fig. 1A). The effects of EASIX quartiles in the multivariable context are visualized in suppl. Figure 1A.

### Validation of EASIX as an independent prognostic marker to predict overall survival after cardiac catheterization

In the EASIXval cohort, blood samples were taken at a median of 174 days (range 28–4246) after coronary angiography (re-established stable clinical phase). 1934 patients of the independent validation cohort had EASIX values taken at a median of 58 days (range 14–365) before CC. Spearman-Rho correlation coefficients between pre- and post-EASIX values were 0.70 (*p* < 0.001) (suppl. Figure 2). This high intercorrelation permits the use of the independent cohort for validation of EASIX effects irrespective of the time point chosen (before or after CC).

In the multivariable Cox model, EASIXval performed similarly as EASIX (Fig. 1B, suppl. Figure 1B). Log2EASIX predicted OS independently (HR: 1.53, CI 1.42–1.64, *p* < 0.001). In both cohorts, EASIX and EASIXval predicted OS independently in heart failure subgroups (LVEF ≥ 50%, LVEF 41–49% and LVEF ≤ 40%), adjusted to age, sex, diabetes, arterial hypertension and a high-grade coronary artery stenosis (in any segment ≥ 90%) (suppl. Figure 3).

The EASIX models were validated by building a new model in the validation cohort with the offset of the prognostic index that was calculated in the training cohort. A comparative estimate near 0 has a similar predictive function in the validation cohort compared to the training cohort. All parameters showed a similar prognostic effect in both models, whereas EASIX and LVEF ≤ 40% showed a slightly better discriminative effect in the validation cohort (estimate above 0) and male sex, CAD and age a slightly worse effect (estimate under 0) (log2EASIX: estimate: 0.17, CI 0.10–0.24 *p* < 0.001, diabetes: estimate − 0.11, CI − 0.29–0.007, *p* = 0.217, hypertension: estimate: 0.09, CI − 0.17–0.34, *p* = 0.501, CAD: estimate: − 0.24, CI − 0.43 to − 0.06, *p* = 0.009, male: estimate: − 0.21, CI − 0.41–0, *p* = 0.047, age: estimate: − 0.07: CI − 0.08 to − 0.06, *p* < 0.001, rEF: estimate: 0.20, CI 0.01–0.38, *p* = 0.036) (suppl. Table 1).

**Fig. 2:**
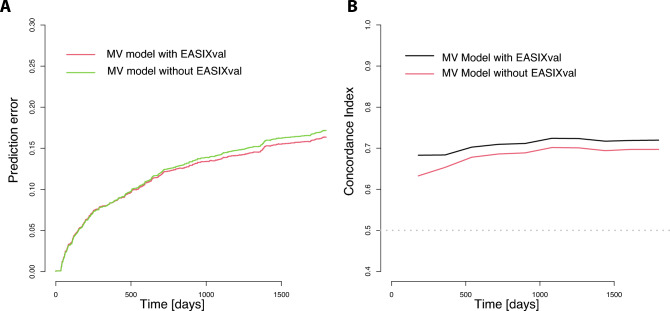
**A** Prediction error curves for 5-year overall mortality (Brier Score) estimated in the validation cohort based on the trained Cox multivariate model from the training cohort (diabetes, hypertension, age, male sex, CAD with any stenosis ≥ 90% and LVEF ≤ 40%) with and without log2EASIX. **B** Concordance indexes in the validation cohort based on the trained Cox multivariate model from the training cohort with and without log2EASIX

The predicted survival probabilities in the validation cohort (EASIXval) with the use of a trained multivariable Cox model in the training cohort (EASIX) including EASIX had lower prediction errors in integrated Brier score analyses (Fig. 2A), and a higher concordance index (Fig. 2B) as compared to models without EASIX. This validates EASIX as an independent predictor of survival in CAD patients.

### EASIX correlates with hs-cTnT and NT-proBNP

**Fig. 3:**
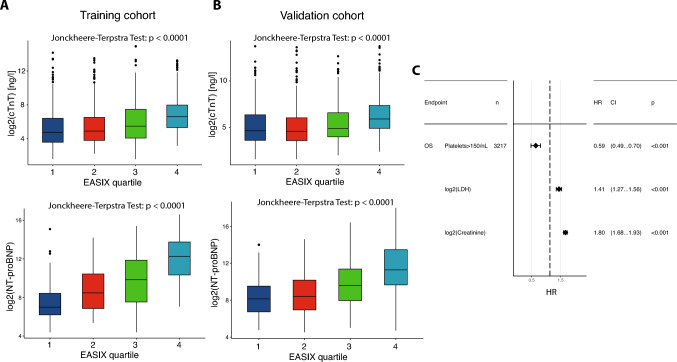
**A** Boxplots showing log2(hs-cTnT) and log2(NT-proBNP) values in dependence of EASIX quartiles in the training cohort. **B** Boxplots showing log2(hs-cTnT) and log2(NT-proBNP) values in dependence of EASIXval quartiles in the validation cohort. **C** Forest plot representing Cox multivariate logistic regression model for 5-year overall mortality in the total cohort (training & validation cohort together). The Hazard ratio for 5-year overall mortality of platelets with the cut-off > 150/nL and continuous log2LDH and log2Creatinine is shown, respectively. *CI* confidence interval, *HR* Hazard ratio, *OS* overall survival

Routine measurements of hs-cTnT and NT-proBNP were performed at the same time as the CC. Figure 3A, B shows that EASIX and EASIXval correlate with both markers at coronary intervention, but strongest with NT-proBNP.

### Single EASIX parameters and overall survival

The impacts of the single EASIX parameters LDH, creatinine and platelets were tested in a multivariable Cox regression including these three markers. As only 291 patients in both cohorts suffered from thrombopenia (< 150/nl), both cohorts were combined and platelets were used as a binary marker (cut-off 150/nl, i.e., lower normal range). Similar to published studies, high LDH and high creatinine together with low platelets were associated significantly with lower survival (Fig. 3C).

### Validation of external cut-offs for EASIX

**Fig. 4:**
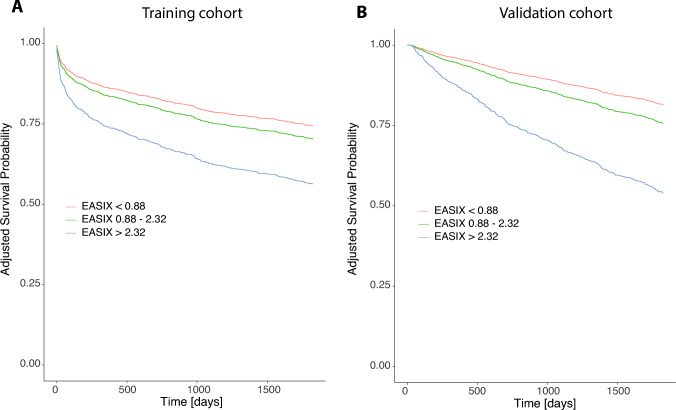
Kaplan–Meier Survival analysis for 5-year overall mortality, using externally validated clinical cut-offs of EASIX (0.88–2.32), adjusted to diabetes, hypertension, male sex, age, CAD (any stenosis ≥ 90%) and a reduced LVEF ≤ 40% in **A** the training cohort (EASIX) and **B** the validation cohort (EASIXval)

Two external cut-offs of EASIX > 0.88 (3.IQR of healthy patients, *n* = 47) and EASIX > 2.32 (previously validated to predict risk of sepsis) were now tested in the multivariable Cox models. Both cut-offs distinguish patients with higher and lower overall mortality in the training and validation cohort (Fig. 4), whereas patients with EASIX values > 2.32 show the highest mortality rates in the two cohorts (suppl. Table 2A, B). These cut-offs may distinguish OS in heart failure subgroups accordingly, even though there are distinct differences in OS between the different categories of LVEF (suppl. Figure 4, suppl. Table 3).

## Discussion

This retrospective cohort analysis establishes EASIX as a potential long-term prognostic marker for patients with coronary artery disease, as a stable marker irrespective of the timing before or after cardiac catheterization. Intra-patient stability of EASIX values was remarkable with a Spearman-rho of 0.7, both before and after CC.

EASIX is built on the routine laboratory marker constellation for TAM after alloSCT, a severe and often lethal complication characterized by increasing LDH and creatinine and declining platelet counts [26]. Although any single marker may associate with a variety of unrelated clinical issues, the coordinated changes of the EASIX ratio allowed a prediction of risk of TAM as early as prior to starting conditioning therapy for alloSCT [27]. EASIX was recently applied as a biomarker to predict mortality in a variety of clinical settings outside transplantation [18, 31, 32, 37–41]. It remained to be established whether EASIX may also help to prognosticate patients with CAD, without severe thrombocytopenia but endothelial dysfunction. The three individual components of the EASIX score LDH [42], creatinine [43–45] and platelets [46] are well established predictors of cardiovascular mortality. Endothelial dysfunction often targets kidney dysfunction (increased creatinine) and associates with increased LDH production by endothelial cells [47]. Interaction of damaged endothelial cells with platelets induces platelet activation and circulating platelet aggregates [48] which increases platelet consumption. The ensuing left shifted platelet production can be visualized by increased numbers of reticulated platelets, a known biomarker strongly associated with mortality in cardiovascular diseases [49].

Patients with ACS or stable angina pectoris, in which macrovascular revascularization is performed, still have an increased mortality rate, with or without secondary ACS events in the following years [50–55]. This suggests a persistence of systemic endothelial and microvascular risks. Endothelial dysfunction, specifically measured via retinal microvascular reactivity to flicker light, was shown to predict long-term patients’ outcome, including major adverse cardiac events (MACE) and OS, in CAD patients [56]. This additionally highlights the importance of assessing endothelial function and its potential in clinical use, over all in long-term cardiac risk stratifications.

Our study includes two independent cohorts of patients with CAD who underwent CC at our institution. As EASIX values before as well as after CC were available in the validation cohort, a Spearman-rho correlation was possible and revealed a high stability of EASIX values over time. However, based on the preselection of patients with only one blood sample before CC, patients with early mortality were assigned to the training cohort. Remarkably, the prediction of long-term outcome based on the EASIX score was not affected.

Moreover, EASIX predicts mortality in CAD patients as a stable marker, independently of rEF (LVEF ≤ 40%) and a macrovascular high-grade coronary stenosis (≥ 90%) at the time of CC. This finding was confirmed in subgroup analysis of different heart failure categories (LVEF ≥ 50%, LVEF 41–49% and LVEF ≤ 40%). CAD and reduced LVEF are parameters that are explicitly treated with medical and interventive treatments. This indicates that the endothelial dysfunction, estimated by EASIX, is an additional, chronic condition in CAD patients that is not, or at least partly, responding to the current treatment options.

EASIX before CC and EASIX after CC both predicted survival and both correlated with NT-proBNP and hs-cTnT at the time of CC. The validation of multivariable models before and after CC was successful in the integrated Brier score and concordance index analyses. This suggests that EASIX contains prognostic information that is independent of CAD. Each of the EASIX components had significant prognostic effects in the multivariable setting, similar to the situation in hematological patients before alloSCT [27]. This again suggests a coordinated alteration of LDH, creatinine and platelets in endothelial dysfunction, similar to the initial observation in TAM.

To bolster the clinical use of EASIX, we validated two externally defined cut-offs to define low EASIX (< 0.88) and high EASIX (> 2.32). We observed a similar hazard ratio (2–3 fold increased risk of death) for high EASIX values in both cohorts.

## Conclusions

EASIX is a potential prognostic marker of survival in patients with CAD, both before and after CC. EASIX combines three laboratory routine parameters that are applicable in any center worldwide. This, together with the validated cut-offs, allows a risk stratification in clinical studies targeting endothelial dysfunction.

## Study limitations

Limitation of our study is the retrospective design and the preselection based on the number of blood samplings, although the two large cohorts and the successful validation of the basal findings should overcome this shortcoming. Due to missing data of other cardiovascular biomarkers, such as NT-proBNP or hs-cTnT, to the same timepoint of the blood draw of the EASIX parameters, we did not evaluate the predictive value of EASIX independently of these established biomarkers. Cardiac biomarkers were not systematically assessed in all patients. This may lead to a selection bias, when analyzing these values. Further, prospective studies are needed to evaluate an additional predictive value of EASIX in comparison to cardiac troponins or NT-proBNP at different timepoints or disease stages.

There are no documented causes of mortality, especially regarding cardiovascular and oncologic causes.

## Supplementary Information

Below is the link to the electronic supplementary material.

Supplementary file1 (PDF 427 KB)
