# Supplementary material for: EASIX (endothelial activation and stress index) predicts mortality in patients with coronary artery disease
Source: Clin Res Cardiol. 2024 Sep 10;114(8):1008–18. doi: 10.1007/s00392-024-02534-y (PMC12283470; doi:10.1007/s00392-024-02534-y)
Supplement: Supplementary file 1 — Supplementary file1 (PDF 427 KB) [file 392_2024_2534_MOESM1_ESM.pdf]

Supplemental Material

EASIX (endothelial activation and stress index) predicts mortality in patients with coronary artery disease

Daniel Finke[1,2], Hauke Hund[1], Norbert Frey[1,2], Thomas Luft[3*#] Lorenz H. Lehmann[1,2,4*#]

[1] *Department of Cardiology, University Hospital Heidelberg, Heidelberg, Germany*
[2] *German Center for Cardiovascular Research (DZHK), partnersite Heidelberg/ Mannheim, Germany*
[3] *Department of Oncology and Haematology, University Hospital Heidelberg, Heidelberg, Germany*
[4] *German Cancer Research Center (DKFZ), Heidelberg, Germany*

# both authors share the last authorship

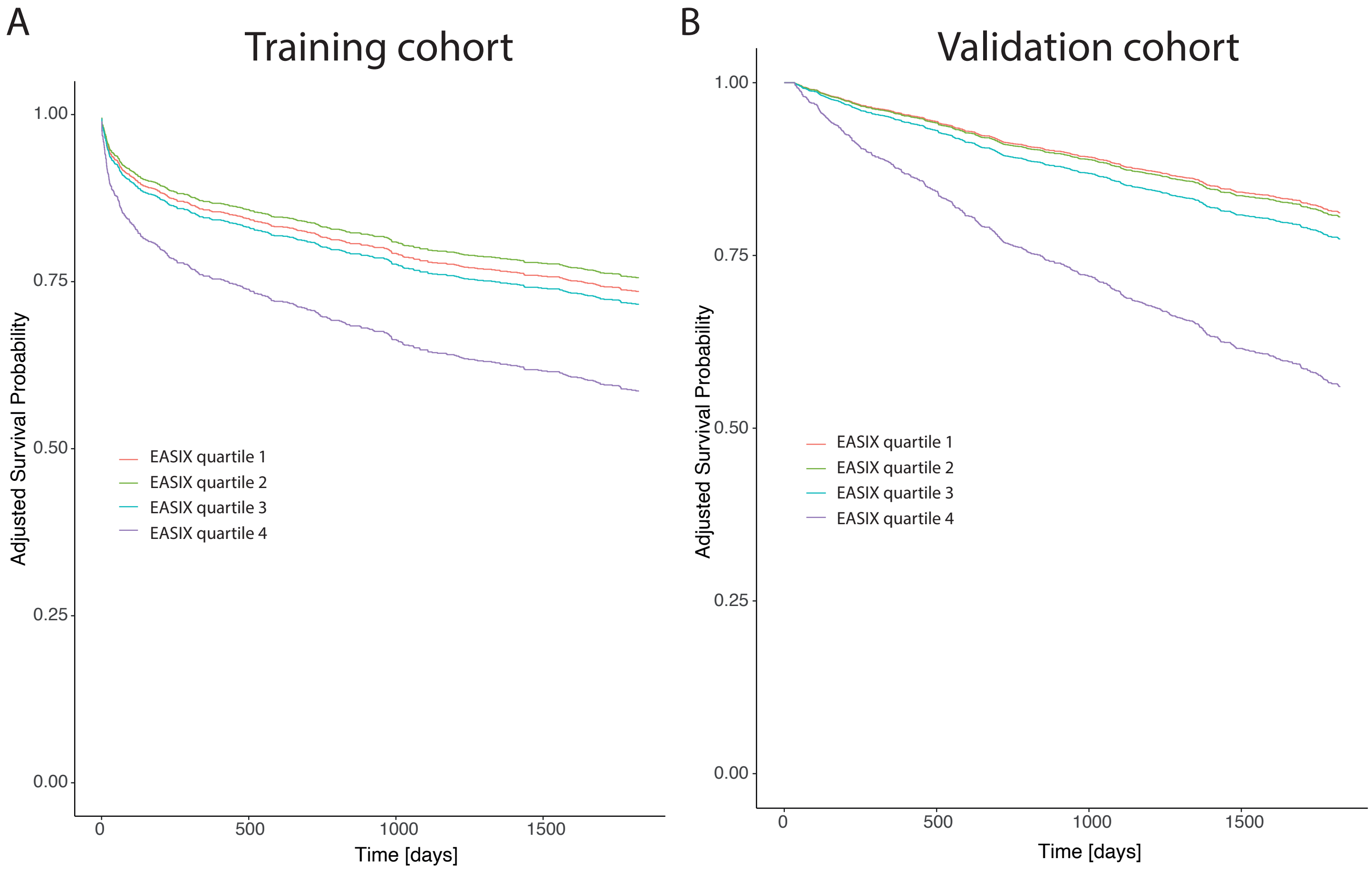

Suppl. Figure 1:
Kaplan Meier Survival analysis for 5-year overall mortality, using EASIX quartiles, adjusted to diabetes, hypertension, male sex, age, CAD (any stenosis ≥ 90%) and a reduced LVEF ≤ 40% in **(A)** the training cohort (EASIX) and **(B)** the validation cohort (EASIXval).

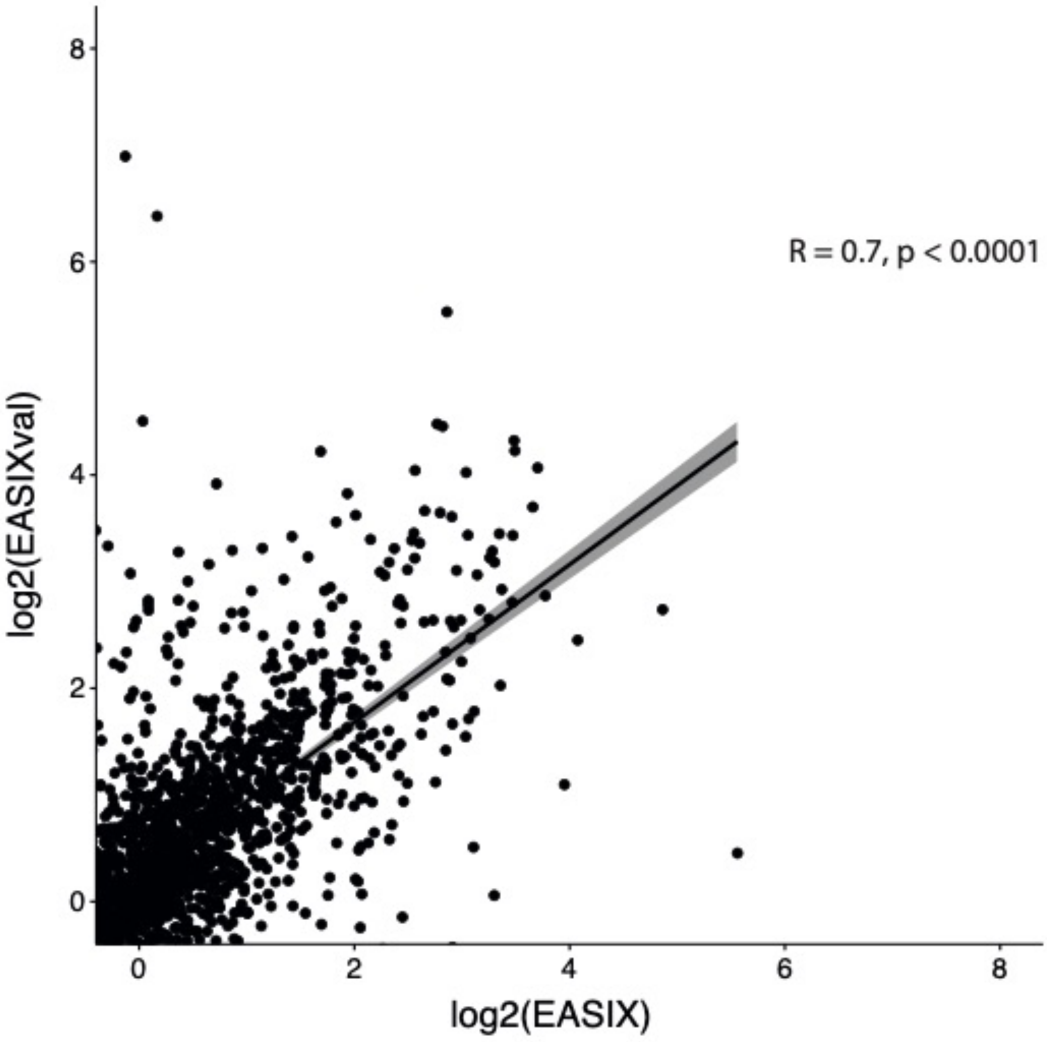

Suppl. Figure 2:
Scatterplot and regression line with 95% confidence interval correlating log2(EASIX) and log2 (EASIXval) in the validation cohort (n = 1934 patients). Spearman's R = 0.7, $p < 0.0001$.

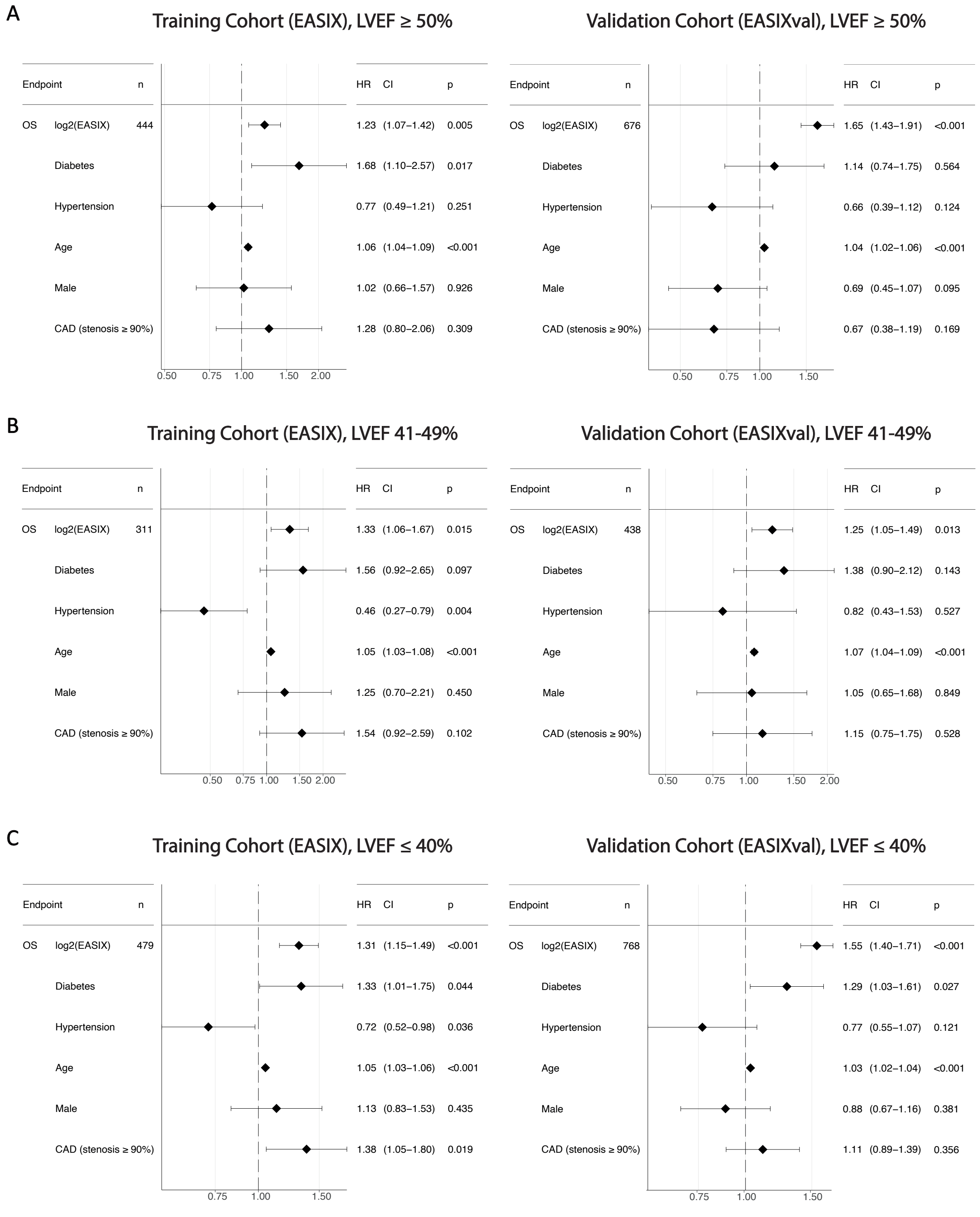

Suppl. Figure 3:
Subgroup analysis of the Cox multivariate logistic regression model for 5-year overall mortality in the training and validation cohort according to heart failure categories **(A)** LVEF ≥ 50%, (**B)** LVEF 41-49% and **(C)** LVEF ≤ 40%, including log2(EASIX), diabetes, hypertension, age, the male sex, a high-grade coronary artery stenosis (≥ 90%), represented in forest plots.

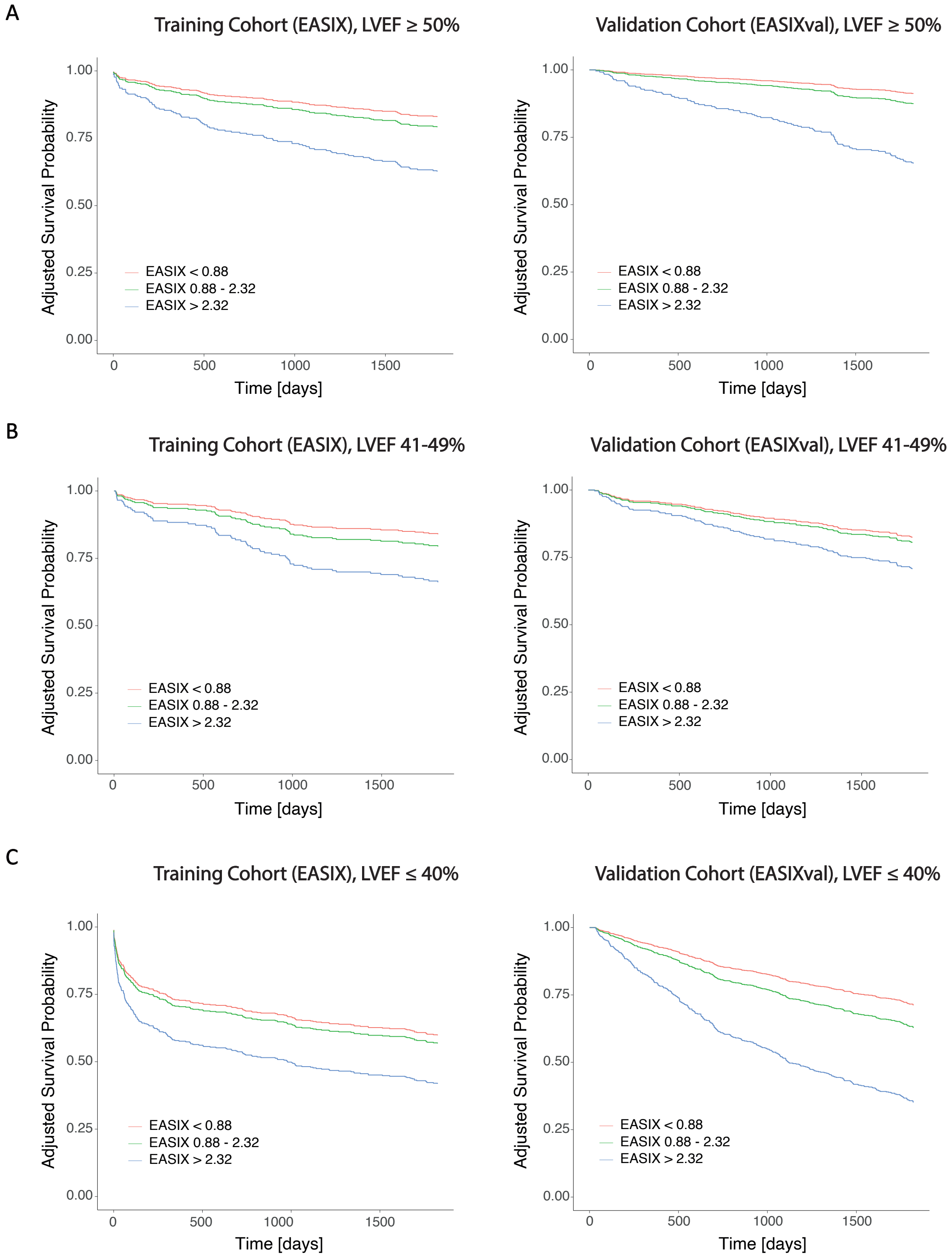

Suppl. Figure 4:
Subgroup analysis of the Cox multivariate logistic regression model for 5-year overall mortality in the training and validation cohort according to heart failure categories **(A)** LVEF ≥ 50%, **(B)** LVEF 41-49% and **(C)** LVEF ≤ 40%, including log2(EASIX), diabetes, hypertension, age, the male sex, a high-grade coronary artery stenosis (≥ 90%), represented in Kaplan-Meier curves, divided by the externally validated cutoffs of 0.88 and 2.32.

Predictors	*Estimates*	*CI*	*p*
EASIXval [log2]	0.17	0.10 – 0.24	**<0.001**
Diabetes	-0.11	-0.29 – 0.07	0.217
Hypertension	0.09	-0.17 – 0.34	0.501
CAD 90	-0.24	-0.43 – -0.06	**0.009**
male	-0.21	-0.41 – -0.00	**0.047**
Age	-0.07	-0.08 – -0.06	**<0.001**
rEF	0.20	0.01 – 0.38	**0.036**
Observations	1882		

Suppl. Table 1:
Cox multivariate logistic regression model for 5-year overall mortality in the validation cohort (EASIXval) with the offset of the prognostic index of the training cohort (EASIX). Log2EASIX, diabetes, hypertension, coronary artery disease (CAD) with any high-grade stenosis (≥ 90%), male sex, age and a reduced LVEF ≤ 40% were used for the multivariate approach.
Estimates near 0 have a comparable prognostic effect in both cohorts. Estimates above 0 and a p-value < 0.05 show a better discrimination of overall mortality in the validation cohort, whereas estimates under 0 and a p-value < 0.05 discriminate less.

A

Training cohort

Predictors	*HR*	*CI*	*p*
EASIX 0.88 -2.32 vs < 0.88	1.21	0.94 – 1.57	0.141
EASIX >2.32 vs < 0.88	2.15	1.60 – 2.89	**<0.001**
Diabetes	1.46	1.18 – 1.81	**<0.001**
Hypertension	0.68	0.54 – 0.85	**0.001**
CAD (stenosis ≥ 90%)	1.36	1.10 – 1.68	**0.004**
Age	1.05	1.04 – 1.06	**<0.001**
Male	1.12	0.90 – 1.40	0.311
LVEF ≤ 40%	2.16	1.75 – 2.68	**<0.001**
Observations	1234		

B

Validation cohort

Predictors	*HR*	*CI*	*p*
EASIX 0.88 -2.32 vs < 0.88	1.38	1.08 – 1.77	**0.010**
EASIX >2.32 vs < 0.88	3.33	2.56 – 4.35	**<0.001**
Diabetes	1.35	1.13 – 1.61	**0.001**
Hypertension	0.72	0.56 – 0.93	**0.013**
CAD (stenosis ≥ 90%)	1.06	0.88 – 1.27	0.557
Age	1.04	1.03 – 1.05	**<0.001**
Male	0.88	0.71 – 1.08	0.208
LVEF ≤ 40%	2.63	2.19 – 3.17	**<0.001**
Observations	1882		

Suppl. Table 2:
(A) Cox multivariate logistic regression model for 5-year overall mortality comparing two EASIX intervals given externally validated cut-offs: EASIX < 0.88 vs EASIX 0.88 -2.32 or EASIX > 2.32, respectively. The multivariate model is adjusted for diabetes, hypertension, age, the male sex, a high-grade coronary artery stenosis (≥ 90%) and reduced LVEF ≤ 40% as confounders in the training cohort **(A)** and the validation cohort **(B)**. CI: confidence interval, HR: Hazard ratio, OS: overall survival

		EASIX			EASIXval			
	Overall	LVEF ≥ 50%	LVEF 41-49%	LVEF ≤ 40%	LVEF ≥ 50%	LVEF 41-49%	LVEF ≤ 40%	p-value
**n**	3217	444	311	479	676	438	768	-
**Sex (male, %)**	2274 (70.7)	254 (57.2)	221 (71.1)	349 (72.9)	451 (66.7)	318 (72.6)	603 (78.5)	<0.001
**Age (median [IQR])**	71 [62, 78]	70 [61.75, 77]	70 [61, 77]	73 [62.5, 81]	69 [60.75, 76]	7.1 [62, 78]	71 [61, 78]	<0.001
**Diabetes (%)**	1083 (33.7)	108 (24.3)	73 (23.5)	161 (33.6)	193 (28.6)	163 (37.2)	340 (44.3)	<0.001
**Hypertension (%)**	2678 (83.2)	329 (74.1)	237 (76.2)	372 (77.7)	592 (87.6)	380 (86.8)	678 (88.3)	<0.001
**BMI < 25 kg/m^2 (%)**	567 (36.7)	61 (34.3)	42 (33.3)	50 (31.1)	164 (38.7)	82 (36.1)	150 (38.9)	0.442
**BMI 25-30 kg/m^2 (%)**	608 (39.3)	76 (42.7)	47 (37.3)	69 (42.9)	175 (41.3)	86 (37.9)	138 (35.8)	0.435
**BMI > 30 kg/m^2 (%)**	371 (24.0)	41 (23.0)	37 (29.4)	42 (26.1)	85 (20.0)	59 (26.0)	98 (25.4)	0.223
**CAD (stenosis ≥ 50%)**	2616 (81.3)	329 (74.1)	258 (83.0)	413 (86.2)	504 (74.6)	363 (82.9)	658 (85.7)	<0.001
**CAD (stenosis ≥ 90%)**	1150 (35.7)	88 (19.8)	109 (35.0)	227 (47.4)	158 (23.4)	154 (35.2)	377 (49.1)	<0.001
**PCI (%)**	1012 (31.5)	115 (25.9)	116 (37.3)	161 (33.6)	213 (31.5)	138 (31.5)	226 (29.4)	0.018
**preCreatinine (median [IQR])**	0.99 [0.81, 1.27]	0.9 [0.75, 1.09]	0.91 [0.80, 1.15]	1.1 [0.84, 1.42]	0.96 [0.80, 1.17]	0.97 [0.83, 1.20]	1.1 [0.89, 1.46]	<0.001
**preLDH (median [IQR])**	240 [200, 290]	230 [198, 275.25]	240 [202, 304]	270 [218.5, 354]	220 [192, 264.25]	230 [195.00, 277.75]	250 [208.00, 299.00]	<0.001
**prePlatelets (median [IQR])**	230 [186, 280]	230 [194, 286]	240 [194, 290.5]	230 [181.5, 290.5]	230 [189, 278.25]	220 [188.25, 268.75]	220 [177.00, 272.00]	0.001
**postCreatinine (median [IQR])**	1.0 [0.86, 1.39]	-	-	-	0.98 [0.83, 1.22]	1.0 [0.83, 1.30]	1.1 [0.94, 1.58]	<0.001
**postLDH (median [IQR])**	240 [203, 291]	-	-	-	230 [197.75, 279.25]	240 [202.25, 282.75]	250 [210.00, 301.00]	<0.001
**postPlatelets (median [IQR])**	220 [181, 270]	-	-	-	220 [186.00, 271.50]	220 [183.00, 272.00]	220 [172.00, 264.25]	0.068
**preEASIX (median [IQR])**	1.1 [0.74, 1.70]	0.9 [0.64, 1.36]	1.0 [0.73, 1.57]	1.4 [0.89, 2.27]	0.96 [0.69, 1.34]	1.0 [0.73, 1.52]	1.3 [0.89, 2.09]	<0.001
**postEASIX (median [IQR])**	1.2 [0.80, 1.89]	-	-	-	1.1 [0.74, 1.56]	1.1 [0.76, 1.74]	1.4 [0.93, 2.21]	<0.001
**hs-cTnT (median [IQR])**	38[16.90, 129.68]	24 [13, 74]	45 [18, 164]	79 [30.09, 373.12]	23 [12, 68.75]	33 [15.00, 82.75]	47 [22, 185.5]	<0.001

	EASIX				EASIXval			
	**Overall**	**LVEF ≥ 50%**	**LVEF 41-49%**	**LVEF ≤ 40%**	**LVEF ≥ 50%**	**LVEF 41-49%**	**LVEF ≤ 40%**	**p-value**
**NT-proBNP (median [IQR])**	580 [169, 2454]	180 [90.5, 713]	270 [126, 783]	4800 [1141, 12338]	300 [118.5, 877.75]	400 [142, 1168]	2300 [733.25, 7605.5]	<0.001
**Median survival (mean (SD))**	1700 (1367.66)	1600 (1329.61)	1400 (1168.16)	950 (1199.86)	2300 (1314.9)	2200 (1507.23)	1600 (1240.27)	<0.001
**5-year overall mortality (%)**	923 (28.7)	1600 (534.97)	1600 (519.19)	1200 (803.54)	1700 (366.01)	1600 (471.73)	1400 (617.05)	<0.001

Suppl. Table 3: Patient characteristics of subgroups according to left ventricular function

BMI body mass index, *CAD* coronary artery disease, *EASIX* endothelial activation and stress index, *hs-cTnT* cardiac high-sensitivity troponin T*, LDH* lactate dehydrogenase, *LVEF* left ventricular function, *NT-proBNP* B-type natriuretic peptide, *PCI* percutaneous coronary intervention, *post* values assessed 28-4246 days after cardiac catheterization, *pre* values assessed 14-365 days before cardiac catheterization
